# Retraction: TOM40 Mediates Mitochondrial Dysfunction Induced by α-Synuclein Accumulation in Parkinson’s Disease

**DOI:** 10.1371/journal.pone.0319935

**Published:** 2025-03-03

**Authors:** The PLOS One Editors

Following the publication of this article [1], concerns were raised regarding results presented in Figs 1, 2, and 3. Specifically,

- There appear to be vertical discontinuities suggestive of splice lines in the following panels:
  ◦ Fig 1A Tom 40, Tom 20, and α-syn
  ◦ Fig 1E Tom 40
  ◦ Fig 2A Tom 40
  ◦ Fig 2C Tom 40
- Lanes 1 and 3 of the Fig 2A Tom 20 panel appear more similar than would be expected for independent results.
- The Fig 3E LV-control α-synA53T and LV-Tomm 40 α-synA30P panels appear to partially overlap.

The corresponding author stated that the underlying data for Figs 1 and 2 are no longer available. In the absence of the uncropped underlying blot data, the image concerns cannot be resolved and the reliability of the associated quantification results cannot be verified.

The corresponding author also stated that the Fig 3E LV-Tomm 40 α-synA30P panel is incorrect. They provided an updated figure and the individual level data underlying the Fig 3B, 3D, and 3F results. These files satisfactorily addressed the Fig 3E concern.

In light of the unresolved concerns with Figs 1 and 2 that call into question the reliability and integrity of the published results, the *PLOS One* Editors retract this article.

AB agreed with the retraction and apologizes for the issues with the published article, but stands by the article’s findings. EM did not agree with the retraction. PD, BS, ER, AA, ME, CL, SM, AOB, MM, EP, and TK either did not respond directly or could not be reached.
